# The Role and Mechanism of Rice Husk Ash Particle Characteristics in Cement Hydration Process

**DOI:** 10.3390/ma17225594

**Published:** 2024-11-15

**Authors:** Jialei Wang, Xiaoqing Hu, Feifei Jiang, Haoyu Chen

**Affiliations:** 1 School of Civil Engineering and Architecture, NingboTech University, Ningbo 315100, China; wangjl@nit.zju.edu.cn (J.W.); chy1416386018@163.com (H.C.); 2 School of Mechanics & Civil Engineering, China University of Mining and Technology, Xuzhou 221116, China; 3 School of Civil Engineering and Architecture, Chongqing Institute of Engineering, Chongqing 400056, China; hu812255201@hotmail.com; 4 School of Civil Engineering, Nantong Institute of Technology, Nantong 226000, China

**Keywords:** rice husk ash, hydration heat, internal curing effect, water-release

## Abstract

Reactive rice husk ash (RHA) is used as a supplementary cementitious material (SCM) to prepare cement composite pastes. The impact of RHA content and the internal curing effect on the hydration process of the cementitious system was studied. The hydration heat, degree, and product content of the cement–RHA composite system at 3, 7, and 28 d were analyzed using hydration microcalorimetry, thermogravimetry, and XRD (Rietveld) analysis. The results show that with the increase in RHA, the main exothermic peaks move forward, and the values increase. The induction period is prolonged, and the acceleration period is shortened. The induction period of 15% RHA is extended to 3 h. The hydration heat of cement composite pastes is mainly divided into three stages. Namely, the first stage (0–18 h) is the superposition of the RHA nucleation effect and chemical effect, the second stage (18–51 h) is the superposition of the dilution effect and internal curing effect, and the third stage (51–72 h) is the internal curing effect with the water-release. The internal curing effect of RHA has a certain periodicity, which is related to its content. The water-release age in the early stage (24 h) advances with the increase of content, and the water-release effect in the later stage (7–28 d) is also significant with the increase of content. The higher the content, the more significant the promotion of the internal curing effect on cement hydration and the pozzolanic reaction of RHA.

## 1. Introduction

Improving the comprehensive utilization rate of solid waste resources is of great significance for environmental improvement and promoting comprehensive green development in society. As a category of solid waste, crop straw is widely sourced and generated in large quantities. Rice husk is one of the main high-yield crop straws, with enormous raw material capacity at a global level. Due to its high silica content, rice husk is difficult to degrade naturally, and the traditional utilization rate is low. If it is used as garbage for landfill, it will increase additional land occupation and soil pollution, which will cause potential harm to the environment. However, energy such as heat and electricity can be generated by burning rice husks. The residual ash, namely RHA, can be used as SCM in cement-based materials, which will greatly increase their added value [1]. The RHA obtained under certain calcination conditions contains a large amount of amorphous SiO_2_ porous powder [2,3]. Its particles can provide nucleation sites for cement hydration, promote the reaction of cement, and improve the hydration degree of cement [4]. The C-S-H gel formed by the pozzolanic reaction further fills the pores and improves the compactness of the pastes. The redistribution of water by the porous structure of RHA can promote the continuous hydration reaction of cementitious materials, as well as the development of mechanical and long-term properties [5,6,7]. Therefore, RHA has excellent physicochemical properties, and its feasibility as an SCM has been fully confirmed [8,9]. Currently, RHA has been researched and applied in many countries such as Asia, Africa, South America, and Western Europe, etc. [10,11,12].

However, compared with traditional pozzolanic materials, the reactivity of RHA and the adsorption of water by its porous structure make the hydration process of the cementitious material more complex. The impact of RHA on cement hydration primarily manifests as nucleation effect, dilution effect, chemical effect [13], and internal curing effect [14,15]. The chemical effect and internal curing effect are related to the amount, reactivity, and porous structure of RHA [16]. The relevant literature [17] studied the effect of RHA on the exothermic reaction of cement hydration. Compared with the control group, 25% RHA significantly accelerated the hydration, advanced the main exothermic peak, and shortened the induction period.

Feng [18] obtained the same conclusion that the adsorption of water by RHA reduced the effective water–cement ratio, and the cement hydration entered an earlier acceleration period. Nguyen [19] found that the impact of RHA on the cement hydration process was related to the water–binder ratio and the amount of RHA. With a constant water–binder ratio, increasing the amount of RHA delays the main exothermic peak, which is different from the conclusion mentioned above. This is due to the different methods of incorporating RHA, that is, the internal incorporation method where RHA replaces cement, or the external incorporation method where the amount of cement remains unchanged. Moreover, when the RHA content is 20%, extra peaks appear in the deceleration period of the exothermic curve for cement pastes with water–binder ratios of 0.4 and 0.6, though the cause of the changes is unclear. The research [20] found that the hydration degree of cement blended with RHA increased significantly after 7 d and was higher than that of pure cement. The study suggests that the internal curing effect of RHA promotes cement hydration by releasing water, thereby increasing the hydration degree. However, most of the previous literature had qualitatively analyzed the results based on relevant theories without discussing the roles and effects of RHA’s reactivity and water-release in the various hydration stages of the cementitious system.

This study aims to investigate the influence of RHA’s reactivity and internal curing effect on different stages of the hydration process in cement-based composite materials. The impact of RHA content on the exothermic rate, hydration degree, and product amount in the cementitious system was analyzed by calorimetric test, TG-DTG analysis, and XRD phase quantification. Additionally, the water-release effect of RHA during the hydration process was examined by testing the relative humidity (RH) within the samples. This study seeks to clarify the influence of RHA’s reactivity and internal curing effect on the hydration process of cementitious materials, providing a reference for the mechanical properties and durability of the composite system.

## 2. Experimental

### 2.1. Materials and Characterization

**Figure 1:**
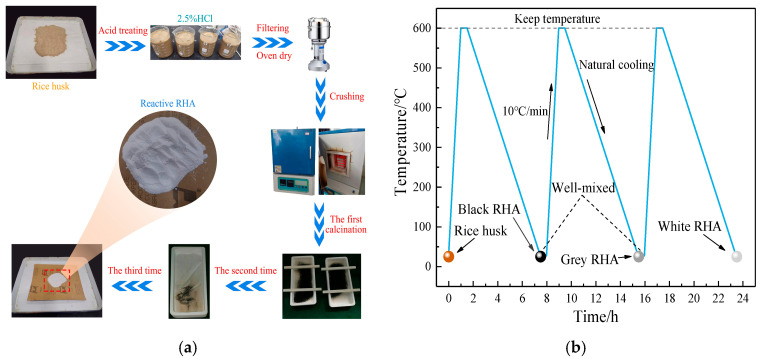
The (**a**) preparation process and (**b**) combustion program of RHA.

**Figure 2:**
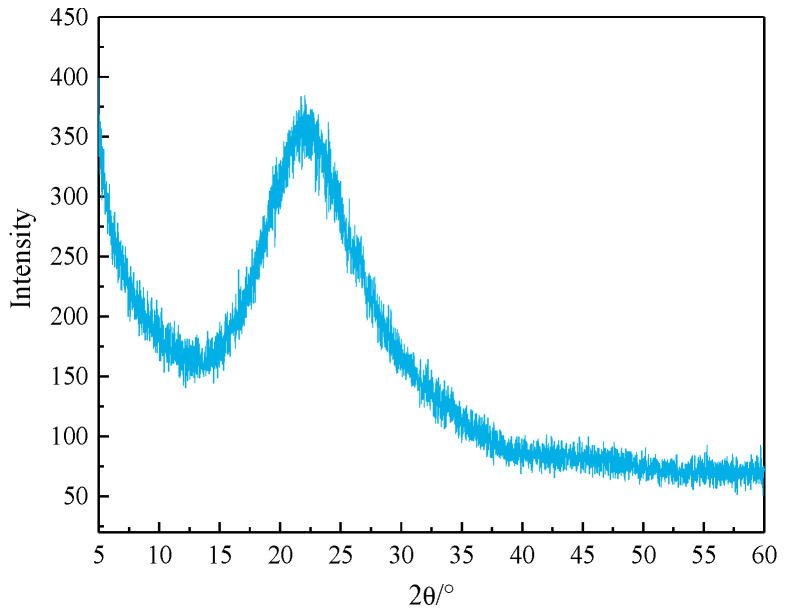
The XRD pattern of RHA.

The cement paste was prepared by P·I 42.5 Portland cement, which was produced by China United Cement Group Co., Ltd, and it was recorded as P. The physical and mechanical properties of cement are shown in Table 1. RHA was prepared according to references as follows [3,21]: rice husks were crushed and finely ground into rice husk powder. A dilute hydrochloric acid solution with a mass concentration of 2.5% was used as a pretreatment agent to soak the rice husk powder at a solid–liquid ratio of 1:10 for 1 h, and the rice husk powder was stirred once every 20 min to ensure thorough acidification. After the pretreatment, the acidified rice husk powder was filtered through an 80-mesh stainless steel sieve, washed, and filtered with clean water 3 times. The acidified rice husk powder was dried in an oven until it formed blocks, then crushed into powder using a crusher. Subsequently, a crucible containing rice husk powder was placed in a muffle furnace for heating, raised from room temperature to 600 °C for 1 h, and maintained for 0.5 h, then the white RHA would be obtained by repeated calcination 3 times, recorded as R. The combustion program and preparation process are shown in Figure 1, and the XRD pattern of RHA is shown in Figure 2. The density of RHA is measured to be 2.21 g/cm^3^.

#### 2.1.1. X-Ray Fluorescence (XRF)

XRF analysis was used to determine the types and content of elements in the powder, which can obtain the chemical composition of RHA. The sample to be tested was ground using an agate mortar, and an Axios mAX type XRF spectrometer produced by PANalytical B.V. in Netherlands was employed for testing, yielding the elemental mass ratio or the mass ratio of oxides in the material. The chemical compositions of cement and RHA are shown in Table 2, and the mineral composition of cement is shown in Table 3. It can be observed that the primary component of RHA is SiO_2_, and a wide peak appears at the position corresponding to the diffraction angle of SiO_2_ in Figure 2, which indicates that the main phase of amorphous SiO_2_ is active in the RHA.

#### 2.1.2. Particle Size Distribution (PSD)

**Figure 3:**
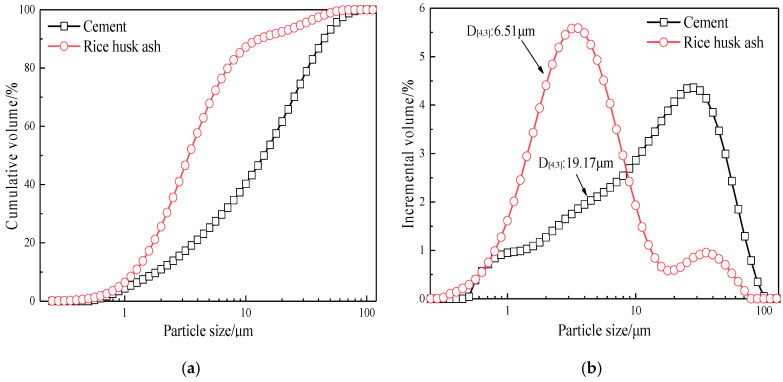
The (**a**) cumulative distribution and (**b**) incremental distribution of cement and RHA.

The PSD test was conducted using a Mastersizer 2000 type laser particle size analyzer produced by Malvern instruments Ltd in UK, which can obtain the particle size. The refractive index of solid particles was set at 0, and the refractive index of the dispersant was set at 1.33, with opacity ranging from 4% to 8%. The testing range was from 0.02 to 2000 μm. Ethanol was used as the dispersant for the testing of cement and RHA. The particle size distribution is shown in Figure 3. It can be observed that the volume average particle diameter of RHA is 6.51 μm, which is smaller than cement.

#### 2.1.3. Scanning Electron Microscope (SEM)

**Figure 4:**
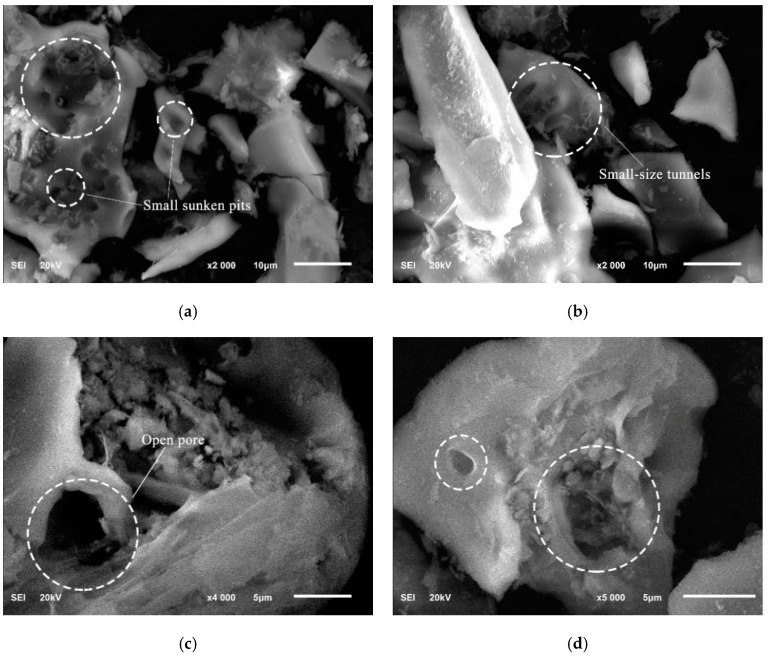
The SEM images (**a**), (**b**) ×2000, (**c**) ×4000, and (**d**) ×5000 of RHA particles.

A JSM-6700F type SEM produced by JEOL in Japan was used to observe the surface morphology and pore characteristics of RHA powder particles. To prevent the powder from being removed under high vacuum and to enhance the conductivity of the powder particles, a gold sputtering treatment was applied to the RHA. The vacuum condition was set to 5 × 10^−5^ Pa, and the operating voltage was 20 kV. The RHA obtained by calcination is a porous powder, where the combustion and separation of organic matter leads to the formation of a porous structure within the RHA particles [13]. The microscopic morphology characteristics of RHA are shown in Figure 4.

It can be seen that RHA particles exhibit polygonal and angular shapes, and the porous surfaces and concave structures represent higher SSA and porosity. The pore structure of RHA particles is mainly characterized by small-sized pits, with some open pores and small-scale tunnels also present.

#### 2.1.4. Porous Structural Characteristics

**Figure 5:**
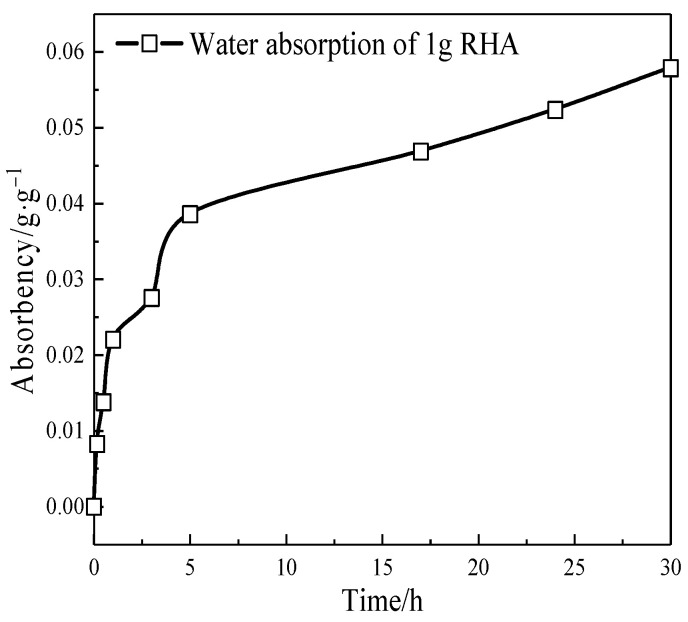
Moisture absorption characteristics of RHA.

**Figure 6:**
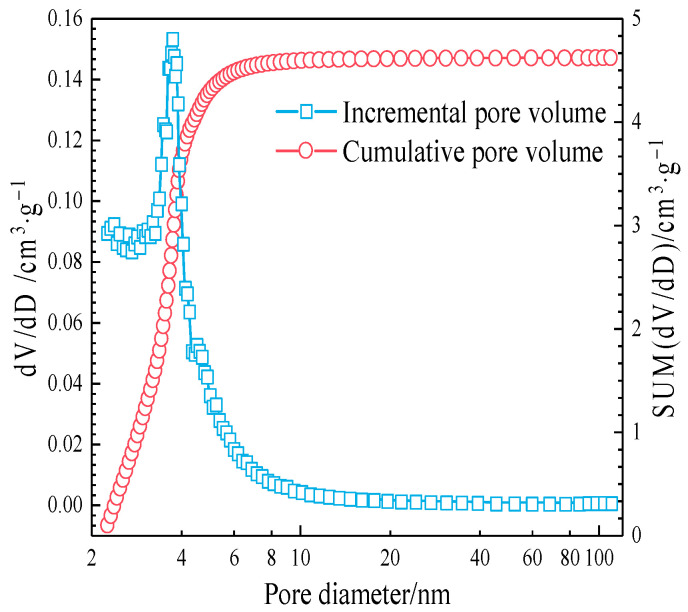
Pore size distribution of RHA.

In order to verify the porous structural characteristics of RHA, the water absorption characteristics of RHA were tested according to the method in references [17,22]. Specifically, the RHA was dried at 110 ± 5 °C for 48 h until a constant weight was reached. A certain quantity of RHA was taken to lay a thin layer in a glass petri dish, which was then placed on an electronic scale and put inside a constant temperature and humidity chamber set at 20 °C and 100% RH. Changes in sample mass with time were recorded, as shown in Figure 5. The specific surface area of RHA was measured to be 207.8 × 10^3^ m^2^/kg using BET-N_2_ adsorption. The pore size distribution of RHA was calculated using the BJH method, as shown in Figure 6. Based on the calculation of the cumulative pore volume per unit mass, the porosity of RHA was 67.04%.

The specific surface area measured by the N_2_ adsorption method typically represents the internal surface area for porous cementitious materials and the external surface area for powder materials. Due to the porous structure of RHA providing more internal surfaces, the measured specific surface area is the sum of the internal and external surface areas. Therefore, the specific surface area of RHA is much greater than that of cement. It can be observed that the micropores of RHA are mainly concentrated in the range of 2–20 nm, as shown in Figure 6.

### 2.2. Experimental Methods

**Figure 7:**
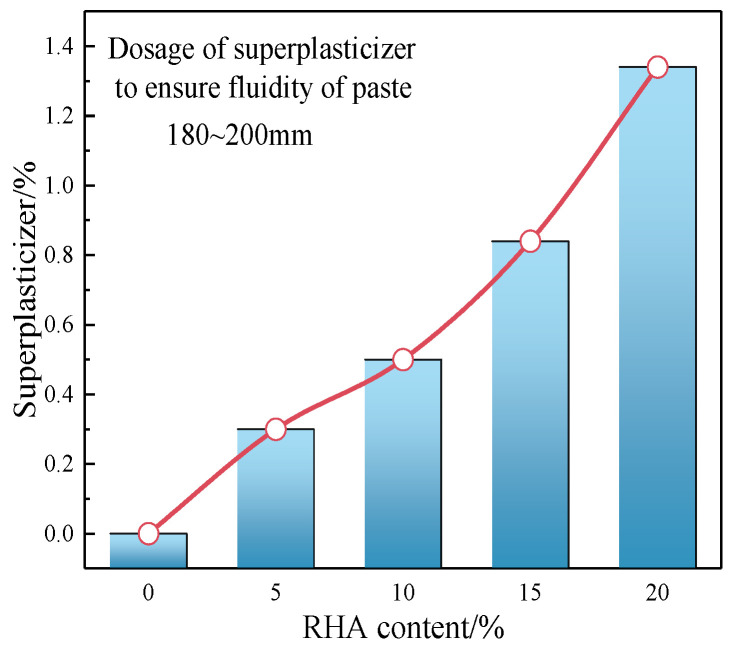
The dosage of superplasticizers required by RHA.

The cement–RHA composite pastes were prepared by using RHA as a substitution for cement in the experimental mix proportions. The water–binder ratio is 0.4, and the RHA replacement rates are 0%, 5%, 10%, 15%, and 20%, respectively, recorded as PC, PR5, PR10, PR15, and PR20. Due to the porous characteristics and high-water storage function of RHA particles, a large dosage of superplasticizer needs to be added when preparing cement composite pastes, as shown in Figure 7. The dosage of superplasticizer with 180–200 mm slump flow of pastes increases with the content of RHA. The more RHA is added, the greater the increase in the superplasticizer dosage. In order to avoid the impact of superplasticizer on the early hydration of cement and ensure the stir-ability and flowability of the pastes, the same dosage (0.4%) can be uniformly used for 5%, 10%, and 15% RHA. The use of 0.4% superplasticizer for 20% RHA pastes cannot be stirred and formed, and excessive superplasticizer may mislead the analysis of hydration heat data. Therefore, the RHA contents for 0%, 5%, 10%, and 15% are selected for the calorimetric test.

#### 2.2.1. Calorimetric Test

TAM Air 8-channel isothermal calorimeter produced by TA instruments Ltd was used to obtain the heat flow curve of cement composite pastes. Samples were prepared outside the calorimeter in an air-conditioned room with a temperature of 20 ± 2 °C. The temperature of the chamber was set at 20 °C. The paste was mixed with an electric stirrer with a rotation speed of 1500 rpm for 3 min and 11.67 g of the sample was weighed for the test. The testing duration is 72 h, with a 10 min interval between the data collection. The calorimetric test of the RHA pozzolanic reaction uses a solid–liquid mass ratio of 1:2, a molar ratio of RHA (SiO_2_) to Ca(OH)_2_ of 1:1, and a testing duration of 50 h.

#### 2.2.2. TG-DTG Analysis

TGA3 thermogravimetric analyzer produced by Mettler Toledo instruments Ltd in Switzerland was used to obtain the TG-DTG curve. Before testing, a portion of the samples was taken after terminated hydration and vacuum drying, then crushed and ground into powder (<100 μm). About 30–40 mg of the powder was placed into a 70 μL corundum crucible, which was inserted into the instrument. The temperature was raised from room temperature to 1000 °C at a rate of 10 °C/min with an N_2_ flow of 20 mL/min. The decomposition temperature range of the phases can be determined from the DTG curves, and the mass loss rate within this range can be obtained from the TG curves. The content of Ca(OH)_2_ and the chemically bound water can be quantified and the degree of cement hydration can be calculated by the TG-DTG analysis.

#### 2.2.3. XRD-Rietveld Analysis

The Rietveld method can achieve quantitative analysis of various phases in the XRD pattern by fitting the whole spectrum of multiple known phases [23]. The sample from the middle position of a crushed specimen was taken and immersed in isopropanol to terminate hydration for 7 d (changing the isopropanol every 3 d). The sample was vacuum-dried in a desiccator for 3 d, ground into powder, and 2 g of the powder was mixed with 0.5 g of analytical pure ZnO (as internal standard). The mixtures were ground for an additional 15 min to ensure uniform mixing, and the particle size was controlled to less than 10 μm by sieving. The mixed powder was then pressed into tablets on a glass slide. The test was performed using a PANalytical X’Pert Pro MPD diffractometer produced by PANalytical B.V in Netherlands, with CuKα radiation, operating at 40 kV and 40 mA, with a scanning speed of 1.56 °/min and a scanning angle (2θ) of 5° to 65°. The quantitative phase analysis was conducted using the internal standard with 20% ZnO. The XRD-Rietveld analysis used X’Pert High Score Plus 3.0e software. The calculation of phase contents is given by Equation (1), while the degree of hydration (DoH) of the cement is provided by Equation (2).

*W*_i_(*t*) = *W*_q_(*t*)/0.8 (1)

(2) $${\left(DoH\right)}_{t}=1-\frac{w_{C_{3}S}(t)+w_{C_{2}S}(t)+w_{C_{3}A}(t)+w_{C_{4}\mathrm{AF}}(t)}{w_{C_{3}S}(t_{0})+w_{C_{2}S}(t_{0})+w_{C_{3}A}(t_{0})+w_{C_{4}\mathrm{AF}}(t_{0})}$$

where *W*_q_(*t*) represents the mass fraction of the phase obtained by XRD–Rietveld analysis; *W*_i_(*t*) denotes the mass fraction of the phase in the cementitious material; *w*_i_(*t*) is the mass fraction of the main phases of cement clinker at the hydration age *t*; *w*_i_(*t*_0_) is the mass fraction of the main phases at the age of initial non-hydration.

#### 2.2.4. Relative Humidity Test

**Figure 8:**
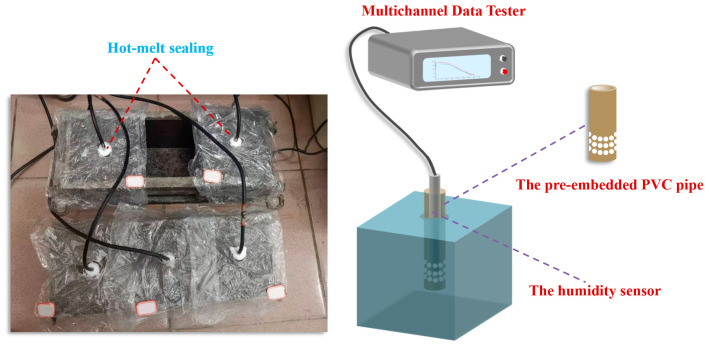
Test of internal RH in the cement–RHA mortar.

In order to understand the water-release effect of RHA, the RH within the paste was measured during the hydration process [24]. A PVC pipe was used to reserve a hole with a diameter of 20 mm and a depth of 80 mm in the center of a 10 cm × 10 cm × 10 cm sample. Multiple pre-drilled holes for the PVC pipe at the central position of the sample were made at a depth of 50 mm (refilled the PVC pipe with filler during mortar forming), which aimed to ensure proper moisture exchange between the sample and the pore, and effectively avoid inaccurate humidity data caused by the sensor contacting with the pastes. To prevent the humidity inside the hole from being affected by the external environment, the reserved hole was sealed with hot-melt adhesive, which also fixed the sensor in place. The outer surface of the sample was sealed with plastic film. The ambient temperature was maintained at 20 ± 2 °C, and the internal RH was recorded every 5 min by using a multichannel data tester, as shown in Figure 8.

## 3. Results and Discussion

### 3.1. The Role of RHA in Hydration Process and Its Mechanism

**Figure 9:**
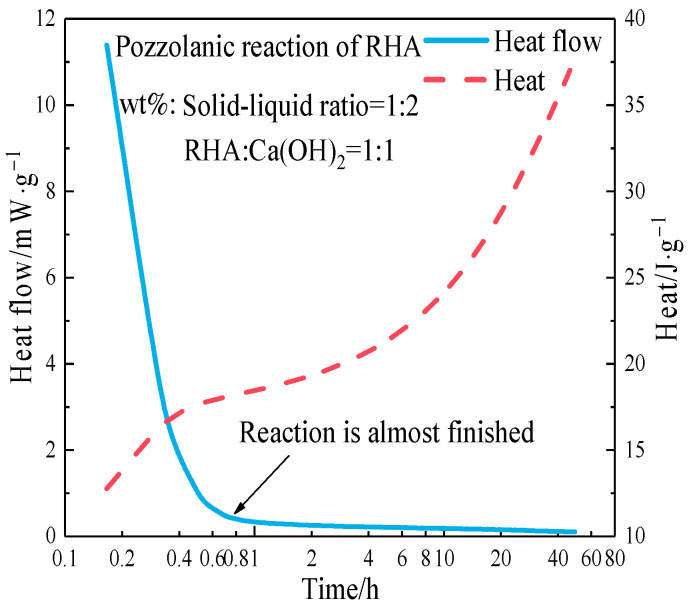
Exothermic curve of pozzolanic reaction of RHA.

**Figure 10:**
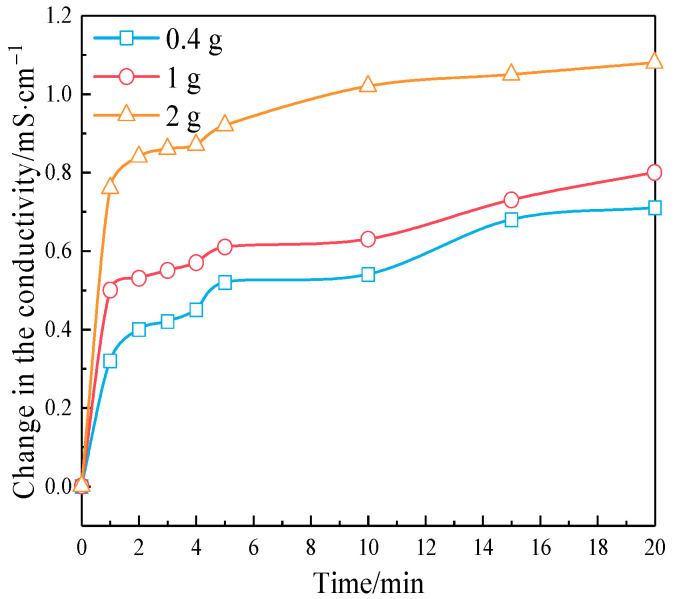
The conductivity variation of saturated Ca(OH)_2_ solution.

Figure 9 shows the exothermic curve of the pozzolanic reaction of RHA. The exothermic reaction rate of RHA decreases rapidly with time and gradually stabilizes after approximately 36 min. The initial reaction already reaches the peak exothermic value of the pozzolanic reaction, which indicates that RHA can react rapidly with Ca(OH)_2_ within a short period. The reactivity of RHA was determined by the conductivity method of saturated Ca(OH)_2_ solution [25]. Figure 10 shows the effect of different RHA masses on the conductivity variation of the saturated Ca(OH)_2_ solution within 0 to 20 min.

**Figure 11:**
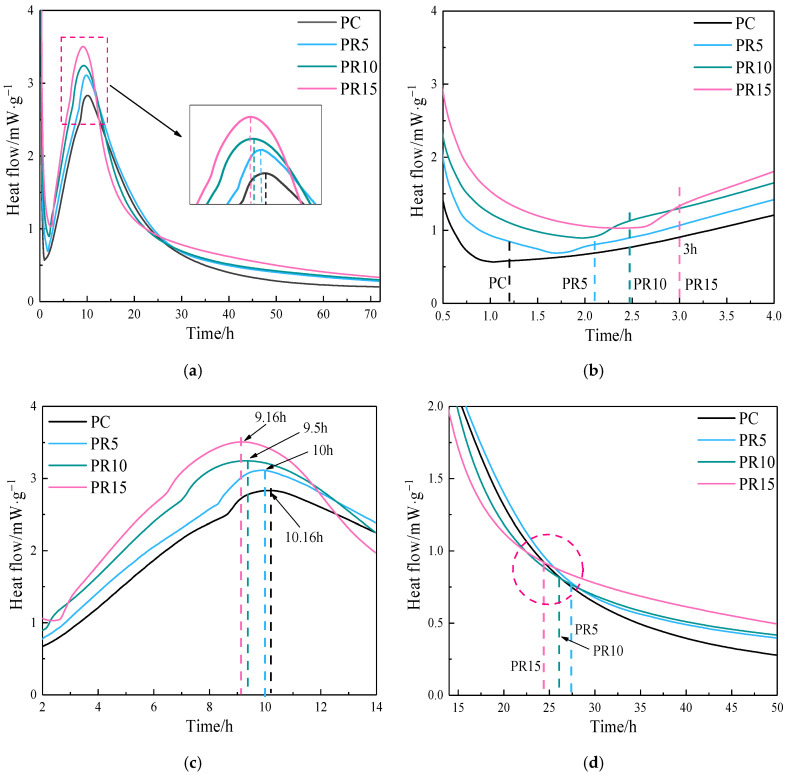
Hydration heat flow (**a**) 0–72 h, (**b**) 0.5–4 h, (**c**) 2–14 h, and (**d**) 14–50 h of cement–RHA pastes.

After adding RHA to saturated Ca(OH)_2_ solution, the amorphous SiO_2_ reacts with Ca(OH)_2_, which reduces the Ca^2+^ concentration in the solution and thereby decreases the conductivity. The greater the variation in conductivity, the higher the reaction activity of RHA. As shown in Figure 9, the RHA quickly produces a pozzolanic reaction in the saturated Ca(OH)_2_ solution, with the reaction being more intense in 0–5 min. After 5 min, the change in conductivity tends to be gentle, indicating a slowdown of the pozzolanic reaction. The increase in the amount of RHA intensifies the reaction degree. It can be seen that RHA has a high reactivity and will react with the Ca(OH)_2_ released by cement hydration in the early stage. However, cement hydration is complicated, and Figure 9 and Figure 10 just represent the reaction degree between RHA and Ca(OH)_2_ in solution, and cannot characterize the start and end times of the pozzolanic reaction in cement paste. Figure 11 shows the effect of RHA on the exothermic reaction rate of the cement paste.

It can be observed that the addition of RHA shifts the main exothermic peak to an earlier time and increases its intensity. The induction period extends with the increase of RHA content, and 15% RHA extends the induction period to 3 h before entering the acceleration period. Figure 11b,c show that the exothermic rate increases with RHA, and the acceleration period delays the start and ends earlier. It indicates that the higher the RHA content, the shorter the acceleration period and the more intense the hydration reaction. During the deceleration period, 10% and 15% RHA reduce the exothermic rate, which is lower than that of the reference group. After reaching the stabilization period (around 24 h), the exothermic rate of the cement paste blended with RHA suddenly changes compared to the reference group and is significantly higher.

**Figure 12:**
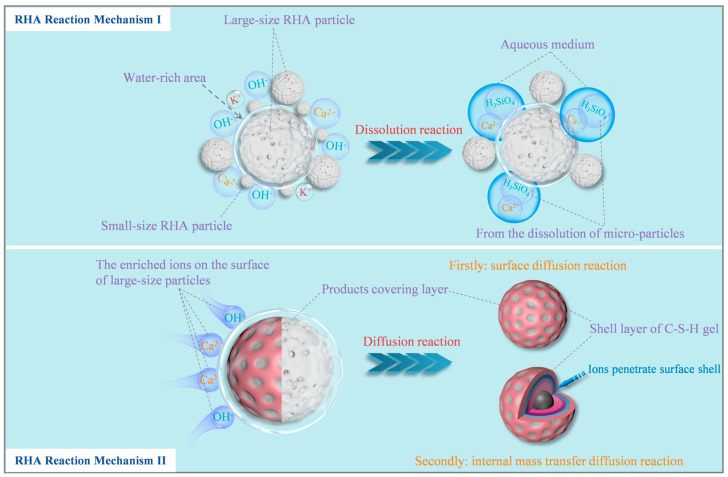
The mechanism of dissolution and diffusion reactions of RHA particles.

Based on the physicochemical properties of RHA particles, it can be clarified that there are two reaction mechanisms of RHA in the cement system [26], as shown in Figure 12, which can fully analyze the influence of RHA on the hydration heat of the composite pastes. Reaction mechanism I: Si-O bonds in RHA are disconnected by OH^−^ ions [27], and hydrolyzed to form the H_3_SiO_4_^−^ ions [28,29], as shown in Equation (3) [30]. The Ca^2+^ ions from the cement hydration react with the H_3_SiO_4_^−^ ions to produce the C-S-H gel. Reaction mechanism II: the surface of partially slow-dissolving RHA particles will be enriched with a large amount of Ca^2+^ ions and OH^−^ ions, which will react with SiO_2_ molecules to form a C-S-H gel layer surrounding the particle surface as the nucleation site [31]. With the processing of hydration, the ions will gradually penetrate through the surface shell and mass transfer to the interior of the particles and continue to the diffusion reaction.

(3) $${\mathrm{SiO}}_{2}(s{)+H}_{2}{O+\mathrm{OH}}^{-}{=H}_{3}{\mathrm{SiO}}_{4}^{-}$$

According to the RHA reaction mechanism, it is known that the hydrolysis of the micro-particles produces H_3_SiO_4_^−^. H_3_SiO_4_^−^ reacts with Ca^2+^, which inhibits the supersaturated crystallization of Ca(OH)_2_, delays the nucleation of Ca(OH)_2_ [32], and prolongs the induction period. Ca^2+^ and OH^−^ released by slowly dissolved C_3_S will gradually accumulate and reach super-saturation, thus leading to the accelerated period, as shown in Figure 11b. Due to the water absorption properties of RHA, the increase of RHA reduces the amount of water available for cement hydration. In the first few minutes of hydration, the amount of dissolved SiO_2_ will decrease. As a result, the induction period is prolonged with the increasing RHA content. Another important reason is that the cementitious system incorporating RHA used an equal dosage of superplasticizer (0.4%). The addition of the superplasticizer can also delay the hydration of the cement and lead to an extended induction period [33].

Due to the filler effect, the addition of any fine particles will increase the reaction rate of C_3_S [34]. The average particle size of RHA is much smaller than that of cement, and the porous structure of the particles provides a large internal and external surface area. This induces the nucleation sites for hydration products to increase with the RHA content. Both the internal and external surfaces of RHA can produce the nucleation effect, facilitating nucleation of the hydration products, and accelerating the hydration reaction of cement and RHA. Thereby, the exothermic rate increases, and the main exothermic peak shifts forward. The earlier end of the acceleration period also indicates that the accelerated hydration reaction causes the rapid nucleation and the growth of hydration products on the surfaces of cement and RHA particles, which encapsulates the unhydrated particles [35], and leads to the beginning of the deceleration period, as shown in Figure 11c.

Around 24 h of hydration, the exothermic rates of the cement paste blended with RHA suddenly changed, as shown in Figure 11d. According to the hydration mechanism of cement [36], the hydration reaction of cement after entering the stabilization period is mainly the diffusion reaction mechanism under the effect of liquid-phase mass transfer. The relevant literature found that the RH inside the samples blended with RHA was higher than that in samples without RHA after 1 d [37]. The water-release of the internal curing effect is the main reason for the higher RH. The release of water promotes cement hydration, and the Ca^2+^ and OH^−^ released by hydrolysis further induce the pozzolanic reaction of RHA, thereby increasing the instantaneous exothermic rate of the composite cementitious system [24].

**Figure 13:**
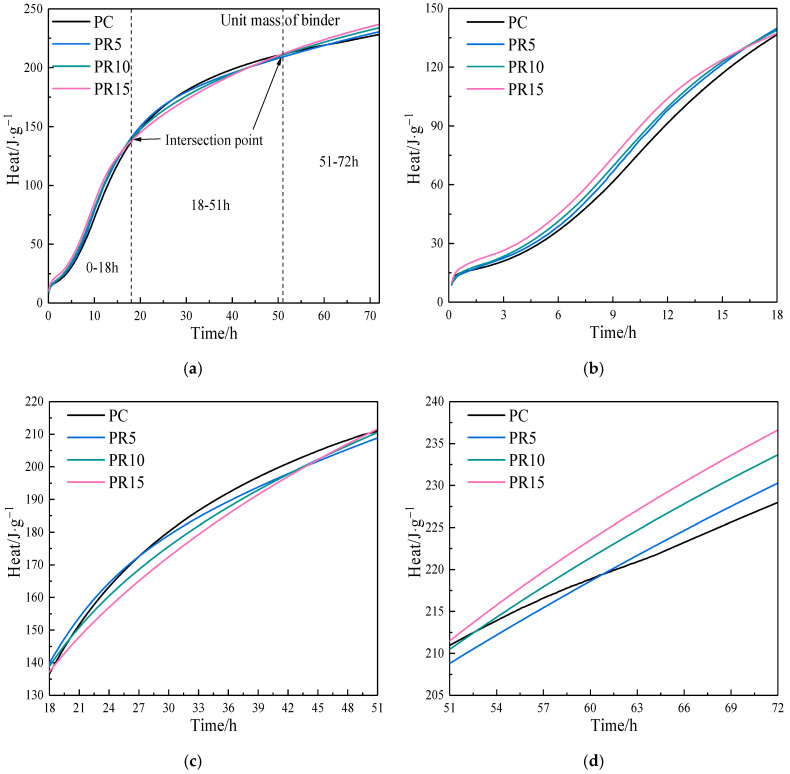
The cumulative heat (**a**) unit mass of binder, (**b**) 0–18 h, (**c**) 18–51 h, and (**d**) 51–72 h of cement–RHA pastes.

The water-release time of internal curing advances with the increase of RHA content. The higher the RHA content, the greater the water storage capacity, and the less water is in the cement paste. The consumption of water by the hydration reaction causes the RH in the system to become unbalanced more quickly, which promotes the RHA to release water earlier. Internal curing of RHA with high content releases relatively more water, which promotes cement hydration and the pozzolanic reaction of RHA more significantly and increases the instantaneous hydration exothermic rate. It can be seen that the water-release time of RHA and the impact on the exothermic rate during 72 hare closely related to the amount of RHA. Figure 13 shows the cumulative heat curves for cement–RHA paste.

Figure 13a shows the cumulative heat of per unit mass cementitious materials. The effect of RHA on the cumulative heat changes twice during the hydration process of the composite pastes, as shown in Figure 13b–d. In the range of 0–18 h, the higher the RHA content, the greater the cumulative heat, which indicates a higher cumulative hydration reaction degree of cement and RHA. The stage includes the initial period, induction period, acceleration period, and deceleration period. The variation in heat release is caused by the superposition of nucleation and chemical effects of RHA. The dissolution of SiO_2_ reduces the OH^−^concentration, and more Ca^2^⁺ ions enter the solution. With the increase of RHA, the dissolution rate of cement particles accelerates after the induction period, resulting in higher heat release. In the range of 18–51 h, there is not only a decrease in cumulative heat caused by the reduced cement content but also an increase in cumulative heat caused by the furtherance of hydration reaction from the water released by RHA around 24 h. This stage is the superposition of dilution and internal curing effects of RHA. The stage of 51–72 h shows an increase in cumulative heat due to the internal curing effect of RHA.

### 3.2. Hydration Degree

**Figure 14:**
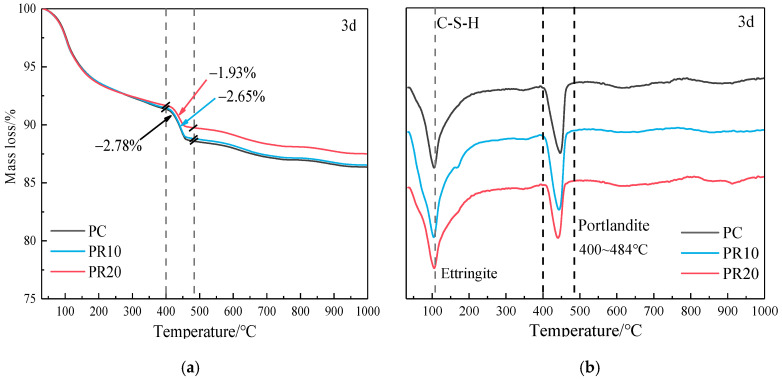

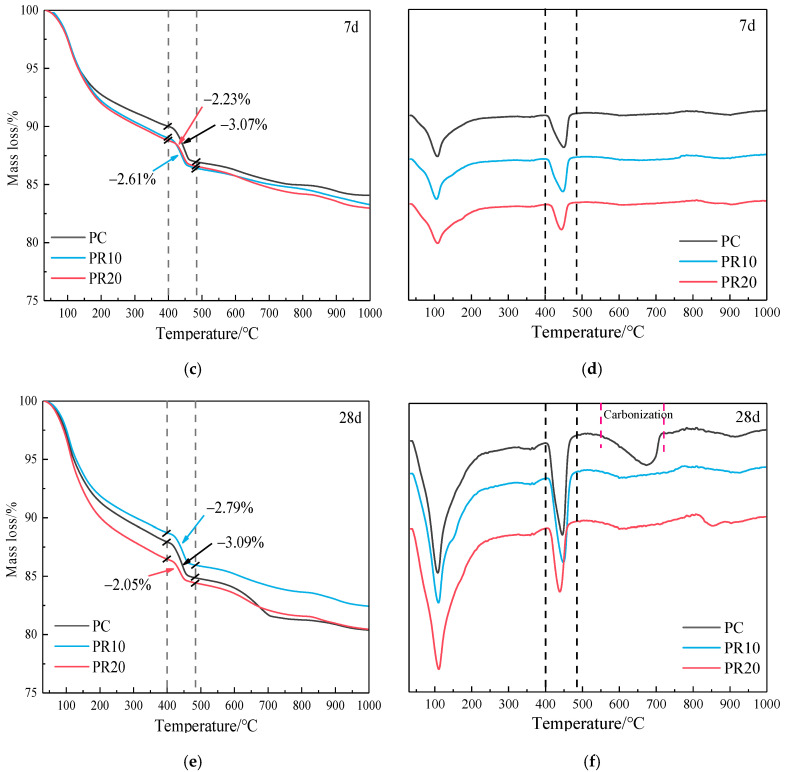
TG-DTG curves of composite pastes hydrated for (**a**,**b**) 3 d, (**c**,**d**) 7 d, and (**e**,**f**) 28 d.

The TG-DTG curves can be divided into two stages [38]: the first stage is the decomposition of hydration products such as C-S-H gels, hydrated calcium aluminate, and AFm phase from room temperature to 300 °C. The second stage is the decomposition of Ca(OH)_2_ and carbonates [39]. The TG-DTG curves of cement–RHA pastes at different ages are shown in Figure 14. The decomposition temperature of Ca(OH)_2_ typically ranges from 400 to 550 °C. However, due to the variations in testing parameters and conditions, the DTG curve should be based on actual testing. From the DTG curve, it can be determined that the decomposition of Ca(OH)_2_ starts at 400 °C and ends at 484 °C. Therefore, 400–484 °C is selected as the temperature range for calculating the Ca(OH)_2_ content. The main mass variation in the DTG curve is the loss of H_2_O below 550 °C.

According to the stoichiometric ratio, 1 mass fraction of Ca(OH)_2_ releases 0.243 mass fraction of H_2_O upon decomposition, which can be used to obtain the Ca(OH)_2_ content. In Figure 14f, the sample of PC hydrated for 28 d shows a mass loss at 550–720 °C, indicating the carbonation of the sample. Based on the molecular weights of CaCO_3_ and Ca(OH)_2_, the sample was corrected to calculate the actual Ca(OH)_2_ content, with a correction factor of 0.595.

**Figure 15:**
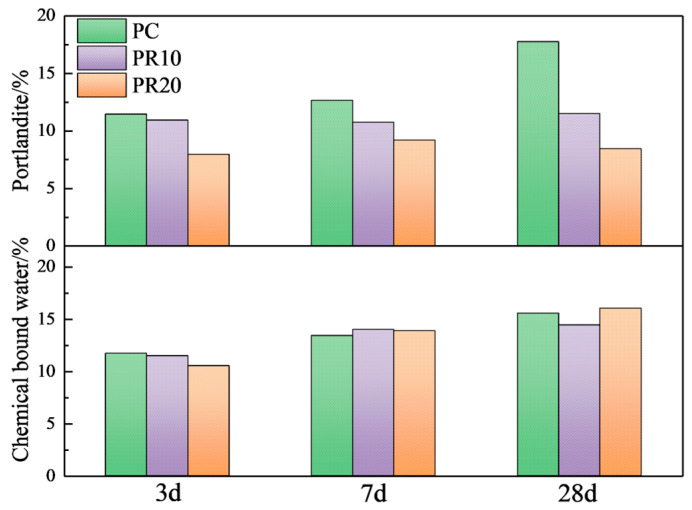
Content of Ca(OH)_2_ and chemical-bound water in cement–RHA pastes at different ages.

The chemical-bound water mainly includes non-evaporable water, the water in hydration products such as AFt, AFm, Ca(OH)_2_, and the water among interlayer pores of C-S-H gels. The amount of chemical-bound water can effectively reflect the content of hydration products to a certain extent. Although RHA will participate in the pozzolanic reaction and consume Ca(OH)_2_, the bound water in the consumed Ca(OH)_2_ will still be present in the C-S-H gel structure [20]. Therefore, the amount of chemical-bound water can accurately reflect the hydration degree of the composite pastes. The phases in the hydration products, except C-S-H, have specific decomposition temperature ranges. The decomposition of C-S-H almost covers the entire temperature range, especially in the range of 100–400 °C [23]. The tested samples were treated with isopropanol to terminate hydration and then vacuum dried, which eliminated the effect of free water loss from capillary pores. The mass loss at 550 °C is selected as the amount of chemical-bound water. The content of Ca(OH)_2_ and chemical-bound water are shown in Figure 15.

From Figure 15, it can be seen that the Ca(OH)_2_ content of PR10 and PR20 is lower than that of PC at all ages, and the reduction in Ca(OH)_2_ content increases with age. The chemically bound water shows different trends. At the age of 3 d, the increase of RHA significantly reduces the Ca(OH)_2_ content, and the chemical-bound water decreases with RHA. At the age of 7 d, the Ca(OH)_2_ content in PR20 shows a slight increase compared to 3 d. With a higher RHA content, the effective water–cement ratio decreases due to the water absorption characteristic, which leads to a decrease in the hydration degree at 3 d. With the progress of the age (3–7 d), a higher RHA content shows a more significant internal curing effect and releases more water, which promotes cement hydration and produces more Ca(OH)_2_. The Ca(OH)₂ content in PR10 at 7 d is slightly lower compared to 3 d, and there is little change in Ca(OH)_2_ content at 7–28 d. The chemically bound water increases and exceeds that of PC at 3–7 d, while the change is minor at 7–28 d. This indicates that the 10% RHA consumes Ca(OH)_2_ through the pozzolanic reaction in the early stage and improves the hydration degree. However, PR20 shows more significant Ca(OH)₂ consumption and hydration promotion from 7 to 28 d. Comparative analysis shows that an appropriate amount of RHA mainly consumes Ca(OH)_2_ and improves hydration in the early stage by the pozzolanic reactions. An excessive amount of RHA mainly shows the internal curing effect and pozzolanic reaction, then promotes hydration of the cementitious system in the later stage.

The chemically bound water comes from the hydration products of cement and RHA. Both the hydration reaction of cement and the pozzolanic reaction of RHA jointly affect the hydration degree of the composite system. Additionally, the water absorption of RHA reduces the amount of water available for cement hydration, impacting the hydration of cement. Due to the multiple factors, the chemically bound water cannot accurately analyze the hydration degree of cement, thus requiring further study combined with XRD quantitative analysis.

### 3.3. Quantitative Analysis of Hydration Products

**Figure 16:**
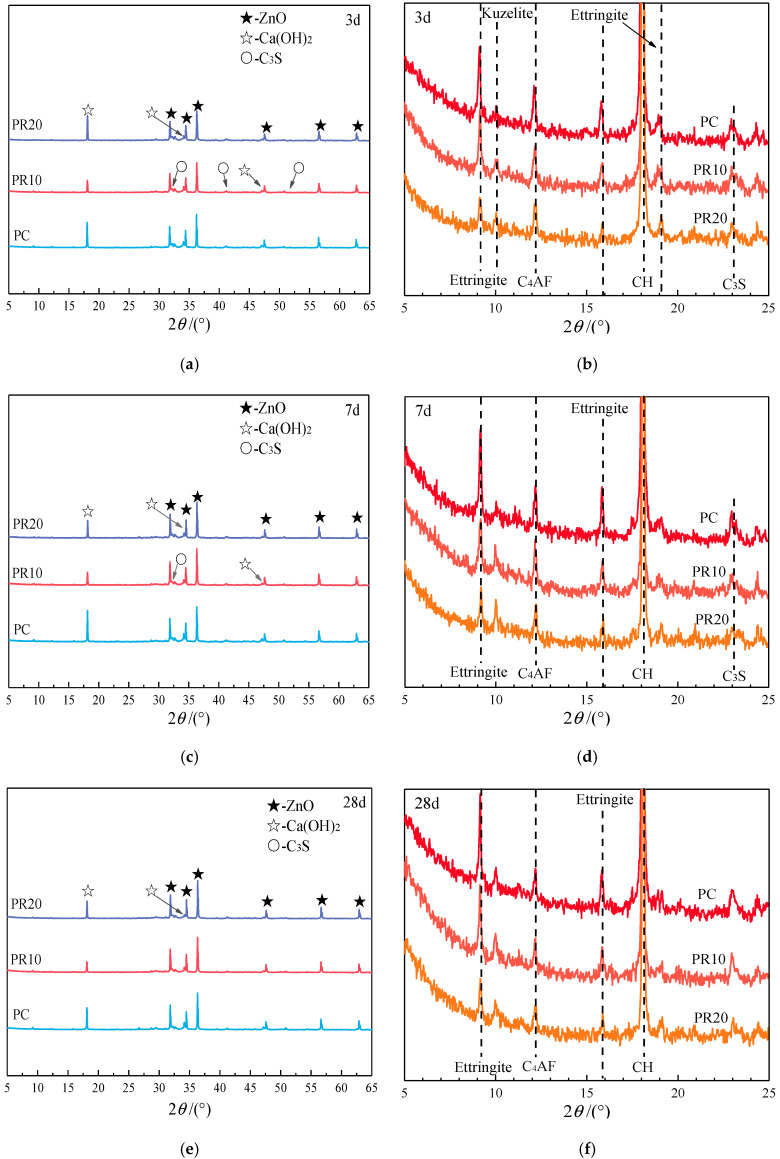
The XRD patterns (**a**,**b**) 3 d, (**c**,**d**) 7 d, and (**e**,**f**) 28 d of cementitious materials at different ages.

In the cement–RHA composite cementitious system, the hydration degree of cement at different ages cannot be determined by TG-DTG analysis. The Rietveld method can be used to quantitatively analyze the content of a single mine in the samples, and then the hydration degree of cement at each age can be calculated. The XRD patterns of the cementitious materials are shown in Figure 16.

From Figure 16, it can be seen that the main phases in the samples are the internal standard ZnO, hydration product Ca(OH)_2_, and the single mine C_3_S. There are no significant differences in phase types with age. The results of the Rietveld quantitative analysis are converted using Equation (1), and the mass fractions of each phase in the cementitious material can be obtained, as shown in Table 4. The early hydration of Portland cement primarily involves the dissolution and precipitation reactions of C_3_S and C_3_A. The C_2_S shows very low reactivity in the early stages, and a relatively obvious hydration reaction appears after 10 d [40]. Thus, the phase content analysis for hydration at 3 and 7 d does not consider C_2_S.

**Figure 17:**
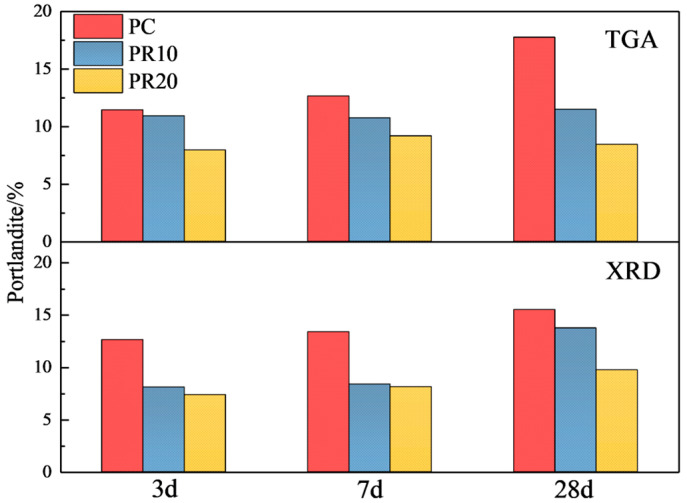
The contrast of Ca(OH)_2_ content in samples measured by XRD and TGA.

Figure 17 compares the Ca(OH)_2_ content obtained from Rietveld quantitative analysis and TG-DTG analysis. It can be seen that a good consistency between the two methods in the Ca(OH)_2_ content changes with the RHA content. According to the single mine content in cement from Table 3 and the calculations in Table 4, the consumption (as a percentage of the cementitious material mass) of single mine at hydration ages of 3, 7, and 28 d is shown in Table 5. The hydration degrees of C_3_S and C_2_S (as a percentage of their own mass) are calculated and presented in Table 6.

**Figure 18:**
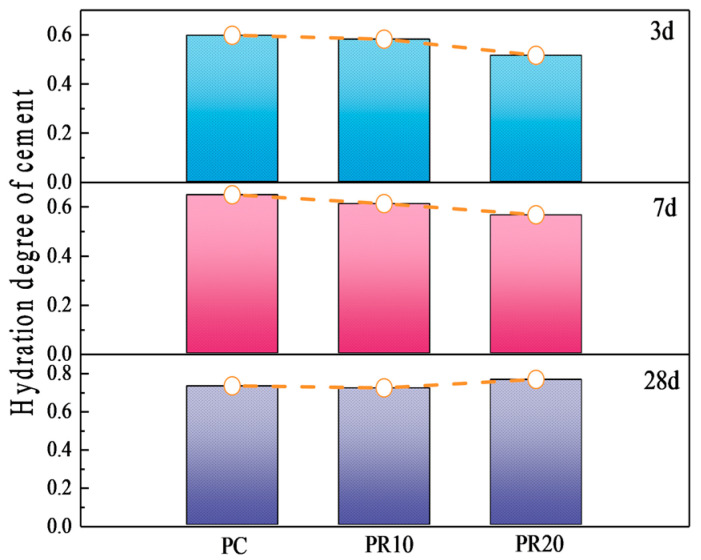
The hydration degree of cement in cementitious material samples.

It can be seen from Table 6 that the higher the content of RHA is added, the more Ca(OH)_2_ is consumed. The hydration degree of C_3_S in samples blended with RHA is lower than that of PC at 3 d and 7 d, and the hydration degree of the cement single mine in PR10 is higher than that of PR20. This indicates that a higher RHA content affects the hydration of cement single mine in the early stage, which is consistent with the previous analysis. The increase of RHA enhances early water absorption, reducing the water available for cement hydration and thereby lowering the hydration degree of single mines. Additionally, the pozzolanic reaction consumes OH^−^ and Ca^2^⁺ released from cement hydration, and H_3_SiO_4_⁻ produced by the hydrolysis of RHA particles will use the surface of cement particles as the nucleation position. This results in pozzolanic reaction products covering the cement particles, causing the cement to enter the diffusion-controlled reaction stage prematurely. The slowing down of cement leads to a decrease in the hydration degree of C_3_S. The Rietveld quantitative calculation was carried out on the cement single mines, and the cement hydration degree was calculated using Equation (2), as shown in Figure 18.

**Figure 19:**
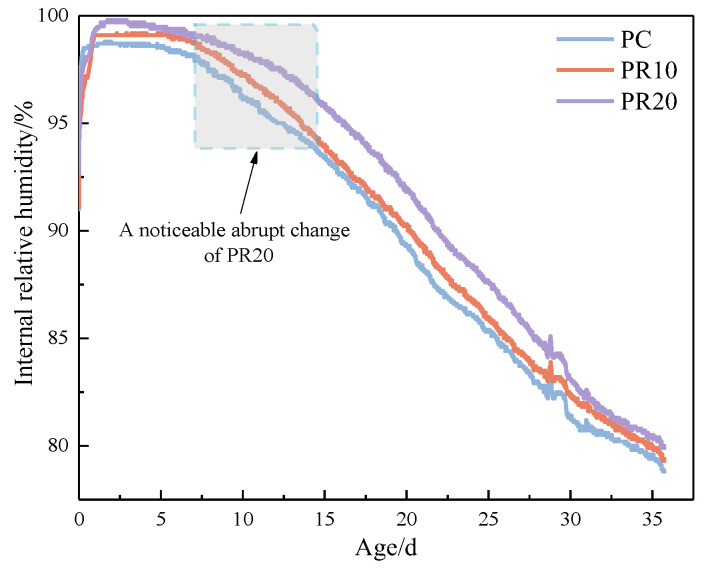
The internal RH of cement–RHA system during hydration.

Figure 18 shows that the cement hydration degree in PR20 significantly increases at 28 d, which is higher than that of other samples. This indicates that more RHA produces water-release at 7–28 d, which facilitates more cement hydration. The Ca(OH)_2_ content obtained by XRD quantitative analysis in Figure 17 is also significantly lower than that of other samples. This suggests that a higher content of RHA promotes the hydrolysis of unreacted C_3_S and C_2_S by the internal curing effect in the later stage of hydration. The released Ca^2^⁺ and OH^−^ migrate to the internal and external surfaces of RHA particles through the pore solution, further stimulating the pozzolanic reactions on their surfaces, thereby improving the hydration degree of the composite cementitious system, which is consistent with the results from Figure 15. The analysis of Figure 11 reveals that RHA produces a water-release around 24 h, and another internal curing effect occurs again at 7–28 d, as shown in Figure 19. The water-release is primarily related to the RH gradient around the particles. With the consumption of free water in the hydration reaction and the evaporation of some free water, a water-release effect is immediately observed when the environmental humidity decreases [37]. It can be seen that the RH in the cement–RHA system remains consistently higher than in the control group after 2 d. This is due to the water-release and moisture-retaining effect of RHA. When the hydration process approaches 7 d, a noticeable abrupt change in RH occurs in samples with 20% RHA, with RH significantly higher than in other groups, effectively alleviating the decline of RH within the cement–RHA system. The pore size distribution of RHA particles ranges from 2–20 nm (Figure 5). Based on Kelvin’s equation, the corresponding humidity range is calculated to be 75–96% [41], and the theoretical humidity is between 96–99% for some larger pores (Figure 6), which means that when the environmental humidity falls below 99%, the water in the RHA pores will be released to compensate for the decrease of humidity. Different environmental humidities will correspond to different degrees of water-release from the pores. In summary, the internal curing effect of RHA exhibits a certain periodicity in the cement–RHA cementitious system, and the higher the RHA content, the more significant the water-release in the later stage.

## 4. Conclusions

(1) With the increase of RHA, the main exothermic peak of the cement–RHA composite paste shifts forward, and the peak value increases. The induction period is prolonged, and the acceleration period is shortened, 15% RHA extends the induction period to 3 h before entering the acceleration period. The water-release time by RHA at an early age and the impact on the exothermic rate are related to the RHA content.
(2) Around 24 h of hydration, RHA produces an internal curing effect, with the water-release occurring earlier as the increase of RHA. The higher the content, the more water-release will be released, and the more significant the promotion of cement hydration and RHA reaction.
(3) The hydration heat of cement composite pastes is mainly divided into three stages. The first stage (0–18 h) is the superposition of the RHA nucleation effect and chemical effect, and the heat increases with RHA. The second stage (18–51 h) is the superposition of the dilution effect and internal curing effect, that is, the reduced heat caused by the decrease of cement and the promotion of hydration from the water-release by the internal curing effect. The third stage (51–72 h) is the internal curing effect with the water-release.
(4) A moderate content of RHA primarily enhances the hydration degree by the pozzolanic reaction in the early stage. The excessive content of RHA mainly contributes to the internal curing effect in the later stage, and also promotes the hydration of the cementitious system by pozzolanic reactions. The water-release of RHA exhibits a certain periodicity, and the higher the content, the more significant the water-release effect in the later stage.

## Figures and Tables

**Table 1:** The physical and mechanical properties of the cement.

Fineness (%)	Density (g·cm^−3^)	Specific Surface Area (m^2^·kg^−1^)	Setting Time (min)		Flexural Strength (MPa)		Compressive Strength (MPa)	
			Initial	Final	3 d	28 d	3 d	28 d
0.6	3.15	349	130	195	5.8	8.8	29.2	50.2

**Table 2:** The chemical compositions of the cement and RHA (wt%).

	SiO_2_	Al_2_O_3_	Fe_2_O_3_	CaO	MgO	SO_3_	Na_2_O_eq_	f-CaO	Cl^−^
Cement	20.75	4.42	3.16	62.59	2.91	2.83	0.53	0.71	0.011
RHA	99.33	0.09	0.05	0.07	0.03	0.08	0.02	0	0

**Table 3:** The mineral composition of cement (wt%).

Minerals	C_3_S	C_2_S	C_3_A	C_4_AF
Cement	56.58	21.76	6.45	10.70

**Table 4:** The quantitative analysis of main phases in samples (wt%).

	3 d			7 d			28 d			
	C_3_S	Ca(OH)_2_	Goodness of Fit	C_3_S	Ca(OH)_2_	Goodness of Fit	C_3_S	C_2_S	Ca(OH)_2_	Goodness of Fit
PC	11.38	12.63	2.08	8.5	13.38	2.07	5.13	16.25	15.50	2.73
PR10	10.69	8.11	1.69	9.67	8.38	1.8	6.62	16.13	13.75	2.04
PR20	12.22	7.38	2.55	9.5	8.13	1.98	3.62	14.32	9.75	2.21

**Table 5:** The consumption amount of C_3_S and C_2_S in samples (%).

Samples	Initial Content		Consumption Amount			
			3 d	7 d	28 d	
	C_3_S	C_2_S	C_3_S	C_3_S	C_3_S	C_2_S
PC	56.58	21.76	45.2	48.08	51.45	5.51
PR10	50.92	19.58	40.23	41.25	44.30	7.45
PR20	45.26	17.41	33.04	35.76	41.64	6.09

**Table 6:** The hydration degree of C_3_S and C_2_S in samples.

Samples	Consumption Amount			
	3 d	7 d	28 d	
	C_3_S	C_3_S	C_3_S	C_2_S
PC	0.80	0.85	0.91	0.25
PR10	0.79	0.81	0.87	0.38
PR20	0.73	0.79	0.92	0.35

## Data Availability

The original contributions presented in the study are included in the article, further inquiries can be directed to the corresponding author.
